# Investigation of α-Klotho Concentrations in Serum of Cats Affected by Hypertrophic Cardiomyopathy

**DOI:** 10.3390/vetsci11050184

**Published:** 2024-04-23

**Authors:** Stephan Neumann, Stephan Siegert

**Affiliations:** Institute of Veterinary Medicine, Georg-August-University of Goettingen, Burckhardtweg 2, 37077 Goettingen, Germany; stephan.siegert@uni-goettingen.de

**Keywords:** α-Klotho, Klotho, feline, cats, hypertrophic cardiomyopathy

## Abstract

**Simple Summary:**

The term Klotho describes a gene and an associated protein that is involved in numerous processes in health and disease (e.g., ageing, kidney damage, cardiovascular disease) and is therefore of increasing interest. Since studies in veterinary medicine are rare, the aim of this study was to investigate Klotho in cats. Serum α-Klotho concentrations were measured in a group of cats affected by a heart disease called hypertrophic cardiomyopathy and a healthy control group. We found no significant difference between the α-Klotho concentrations in the two groups. However, a comparison of the different disease stages provided indications of possible correlations of α-Klotho with disease stages. In veterinary medicine, the assessment of Klotho in heart disease could be of interest for future studies, as it shows promising properties and potential applications in disease therapy and prevention.

**Abstract:**

Being involved in various physiological and pathophysiological mechanisms (ageing, kidney damage, cardiovascular diseases, etc.), Klotho is a parameter of increasing interest. Studies in veterinary medicine are still rare, but it is exciting to find out whether the findings obtained can be transferred to animals. The aim of this study was therefore to investigate Klotho in cats. This study addressed α-Klotho concentrations in the serum of two groups of cats: one diseased group affected by hypertrophic cardiomyopathy (*n* = 27) and one healthy control group (*n* = 35). α-Klotho concentrations in serum were measured using an ELISA. The results were evaluated in the context of several echocardiographic measurement parameters in the diseased group. No significant difference between α-Klotho concentrations in the two groups was found. A slight negative correlation was found between α-Klotho concentrations and the relation of left atrium/aorta (La/Ao) in the diseased group. Gaining initial information on α-Klotho in cats, it was not possible to draw definite conclusions concerning cardiomyopathies in this species. The assessment of Klotho should be considered in terms of its broad implications in disease processes, but it is also recommended to focus on specific disease features. Both approaches might be promising as possible applications of Klotho in veterinary medicine.

## 1. Introduction

Hypertrophic cardiomyopathy (HCM) is considered the most common heart disease in cats and one of the leading causes of death in this species [1,2]. HCM is a primary genetic disease. There is a predisposition for Maine Coon, Bengal, Ragdoll, British Shorthair, Norwegian Forest, Siberian Forest, Persian, Scottish Fold, and Sphynx. The disease is also more common in males [3].

The aetiology of HCM appears to be defective protein synthesis caused by genetic mutations, resulting in a sarcomere defect in the cardiomyocytes. This leads to hypertrophy, i.e., an increase in the size of the cardiomyocytes themselves; a misalignment of the myocytes; and an increase in cardiac mass. In addition, changes in the blood supply to the muscle tissue itself lead to an ischaemic effect associated with fibrosis [4,5]. Mutations in the myosin-binding protein C gene (MYBPC 3) have been identified as a possible cause in Maine Coons and Ragdolls but are unlikely to be the sole cause of the disease [6].

The diagnosis of HCM is currently made by ultrasound after clinical signs or as part of preventive examinations. The same applies to the health monitoring of affected animals. Alternatively, a suitable biomarker could assist in the identification and monitoring of affected animals.

Currently, troponin and NT-proBNP (N-terminal prohormone of B-type natriuretic peptide) are used as cardiac biomarkers. Troponin is a binding molecule between actin and myosin and is therefore functionally involved in myocyte contraction. Accordingly, diseases of the myocytes can release troponin. The conclusion that diseases associated with myocyte necrosis or other reductions in cell membrane continuity result in increased serum tropinin concentrations has led to its use in human and veterinary medicine as a marker of infarction. As this disease is rare in dogs and cats, there are currently few clinical applications of troponin in veterinary medicine [7,8].

The situation is different with NT-proBNP, which is pathophysiologically integrated into the organism’s response to volume overload and, thus, to myocyte “stress”. In the event of volume overload, NT-proBNP is produced in increased amounts, leading to vasodilatation and increased diuresis, resulting in a reduction in volume and pressure. The clinical use of NT-proBNP is primarily in the differentiation of respiratory symptoms of cardiac or non-cardiac origin [9,10]. Other biomarkers for the diagnosis or monitoring of hypertrophic cardiomyopathy have been less explored. In these studies, we looked more closely at Klotho.

Klotho has recently gained increasing attention in science due to its involvement in multiple processes in health and disease. It was described originally in 1997 as a gene regulating ageing [11]. Since then, various functional features of the gene and its corresponding protein have been noted. Its functions include acting as an anti-ageing factor, acting as a regulator of oxidative stress, being associated with aspects of several illnesses like kidney or cardiovascular diseases, having an influence on growth factor signalling, and being a co-receptor for endocrine fibroblast growth factors, among others [12,13,14,15,16]. The term Klotho includes both the mentioned gene and the associated protein, a transmembrane protein consisting of three domains: a cytoplasmatic domain, a transmembrane domain, and an ectodomain [16]. The Klotho gene is mainly expressed in the kidney [11], while the soluble protein reaches several organs through blood circulation. When the Klotho protein is referred to, usually α-Klotho is implied. There are two more members of the Klotho family, namely β- and γ-Klotho [15]. Circulating α-Klotho derives either from release through secretion or through shedding from the Klotho-ectodomain [16]. Mice with defective Klotho gene expression showed slower growing and shortened lifespans, which led to the assumption of Klotho being capable of suppressing ageing phenotypes [11]. In addition to the ageing-related signs, these animals showed arteriosclerosis and ectopic calcification of the cardiac muscle, among other tissues [11]. Semba et al. [17] found an association of the Klotho concentration and cardiovascular diseases independent from other diseases: the risk of cardiovascular disease in human patients was lower with higher plasma Klotho concentrations in this study. Several other studies dealing with Klotho in the context of cardiovascular and concomitant diseases have described various interplays. Klotho was referred to as having cardioprotective features in [18]. Xie et al. [18] stated that Klotho deficiency was associated with the appearance of excessive cardiac hypertrophy in a mice experiment, while it was not generally associated with cardiac pathologies. Klotho and its cardiovascular implications have been described in the context of chronic kidney disease (CKD) in both experimental and clinical contexts too. In human patients with CKD-related left ventricular hypertrophy (LVH), elevated left ventricular mass was related to lower serum Klotho, and the presence of Klotho caused the inhibition of cardiomyocyte hypertrophy induced by uremic toxins in vitro [19]. Olejnik et al. [16] even linked Klotho to therapy options for cardiovascular disease by referring to its ability to positively influence pathological alterations in the heart. In veterinary medicine, Klotho has been described in canine patients with CKD [20] and mammary gland tumours [21] so far. Klotho seems to be a promising parameter in the context of cardiovascular diseases. Therefore, we aimed to expand the knowledge about the association of this parameter and cardiac diseases in companion animals. Hypertrophic cardiomyopathy (HCM), being the most common heart disease in cats [22], with various implications on heart function and health condition, was therefore chosen for analysis in this study.

## 2. Materials and Methods

### 2.1. Animals

A total of 62 cats were examined in this study, of which 27 represent the group affected by HCM (HCM group) and 35 comprised the healthy control group. All cats were client-owned and were presented to the Institute of Veterinary Medicine between December 2018 and October 2021 for routine or diagnostic examination. As diagnostic procedures, they were subjected to a clinical examination and blood analysis (haematology and serum chemistry). Cats with suspected or pre-existing heart disease were also subjected to an echocardiographic examination. Body Condition Score (BCS) (score 1–9) was assessed according to the World Small Animal Veterinary Association (WSAVA) Global Nutrition Committee guidelines [23]. The cats in the healthy control group did not show any signs of disease in their medical records, physical examinations, or blood analyses. The diagnosis for cats with HCM was made based on echocardiography. An increased, diffusely or focally occurring, left ventricular wall thickness (LVWT; end-diastolic wall thickness ≥ 0.55 cm) was detected in these patients, either within the process of routine or control examination or during examination because of acute illness. Other systemic illnesses that can also cause increased LVWT were ruled out, e.g., hyperthyroidism, systemic hypertension, and pseudohypertrophy. To rule out hyperthyroidism, the thyroxine value (reference 0.9–2.9 µg/dL) was determined, and only animals with a value within the reference range were considered in the study. To rule out systemic hypertension, the measurement of systemic blood pressure was carried out at the time of echocardiography.

Potential pseudohypertrophy was addressed by considering the hydration status of the animal, paying particular attention to potential dehydration. All experimental procedures were operated after approval by the regional authorities for Consumer Protection and Food Safety in Lower Saxony (reference number 33.9-42502-05-18A361), Germany, and under the supervision of the university’s Animal Welfare Officer.

### 2.2. Samples

Before any treatment blood samples used for all following measurements were collected from the cephalic vein and depending on the following analysis, blood was put in ethylenediaminetetraacetic acid (EDTA)-prepared tubes or serum separator tubes (both from Sarstedt AG & Co., KG, Nümbrecht, Germany). An analysis of haematology was conducted with a ProCyte Dx Analyzer (IDEXX GmbH, Kornwestheim, Germany), and an analysis of serum chemistry was conducted with a Thermo Scientific Konelab 20i Clinical Chemistry Analyzer (Thermo Fisher Scientific Inc., Waltham, MA, USA). Blood for α-Klotho measurements was collected into serum separator tubes (Sarstedt AG & Co., KG, Nümbrecht, Germany), allowed to clot at room temperature, and then centrifuged at 1000× *g* for 20 min. Afterwards, serum was pipetted into reaction tubes (Sarstedt AG & Co., KG, Nümbrecht, Germany) and stored in aliquots at −80 °C until analysis.

### 2.3. Echocardiography

Echocardiography was performed by a certified veterinary cardiology specialist using an ultrasonic device (CX50, Philips, Amsterdam, The Netherlands) with a sector array transducer with 4–12 MHz (S12-4, Philips, Amsterdam, The Netherlands). The examination was performed on cats in lateral recumbency or standing positions, without sedation, depicting the right parasternal view to record linear measurements. The echocardiographic examination was part of the usual clinical diagnostic or control examination. The parameters for subsequent analysis in this study were taken from the clinical records. These parameters included the following: case history; LVWT, including end-diastolic left ventricular free wall thickness (LVFWd) and end-diastolic interventricular septal thickness (IVSd); left atrial size (La); and ratio of left atrium to aorta (La/Ao). Concerning LVFWd and IVSd, the respective higher values were used for analysis. Based on the above information, the cats were classified with reference to the stages of feline cardiomyopathy as described by the American College of Veterinary Internal Medicine (ACVIM) consensus statement guidelines [24].

### 2.4. Assay

α-Klotho serum concentrations (pg/mL) were measured using a commercially available ELISA kit (Cat Soluble Alpha-Klotho (SAKL), MBS9357669, MyBioSource, Inc, San Diego, CA, USA). This assay was a Sandwich ELISA with 96 wells and a detection range of 6.25–200 pg/mL. The intra- and inter-assay coefficient of variation were <15%, as indicated by the manufacturer. The assay was performed following the manufacturer’s instructions. Standards and samples were applied in duplicate on the ELISA plate. The assay procedure was as follows: Standards and serum samples were placed on the pre-coated microtiter plate, and enzyme-linked reagent was added. The microtiter plate was incubated for 60 min at 37 °C, followed by manual washing. Afterwards, hydrogen peroxide and TMB–substrate were added and incubated for approximately 15 min at 37 °C until the appearance of an adequate colour reaction. Finally, adding an acidic stop solution terminated the enzymatic colour reaction and caused a colour change from blue to yellow. The optical density (O.D.) of each well was determined immediately at a wavelength of 450 nm using a microplate reader (TECAN GENios Pro, TECAN AUSTRIA GmbH, Grödig, Austria). Computer software (CurveExpert Professional 2.6, Hyams Development, https://www.curveexpert.net/) was used to fit a standard curve and to calculate the referring serum concentrations.

### 2.5. Statistical Analysis

Statistical analysis was performed using commercial statistical software (GraphPad Prism version 9.2.0 for Windows, GraphPad Software, San Diego, CA, USA, www.graphpad.com).

Data were tested for normality using the D’Agostino–Pearson, Anderson–Darling, and Shapiro–Wilk tests. Nonparametric methods were used for the comparison of groups: the Mann–Whitney U test was used for the comparison of two groups, and the Kruskal–Wallis test was used for the comparison of multiple groups. Variables are presented as medians (interquartile ranges [IQRs]). The Tukey format was used for the presentation of boxplots. The calculation of the Spearman correlation was performed to analyse the relationship between parameters. A *p* value ≤ 0.05 was considered statistically significant.

## 3. Results

Of the initial 35 samples in the control group, one had to be excluded due to a measurement error. Therefore, the calculations for this group are based on 34 samples. α-Klotho was detectable in the serum samples of both analysed groups. The measured values fell within the lower quarter of the assay’s detection range. For the descriptive statistics results, see Table 1.

**Figure 1:**
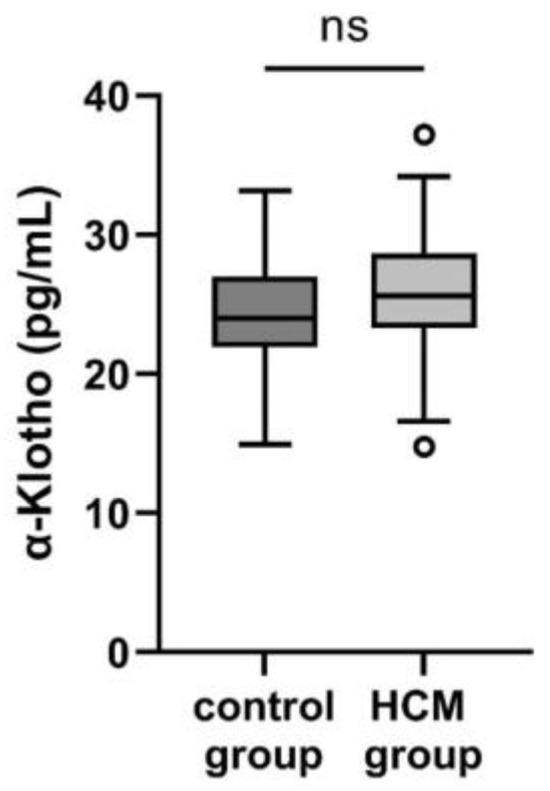
α-Klotho concentration in control group and HCM group as group comparison. Legend: ○ outlier, ns not significant.

**Figure 2:**
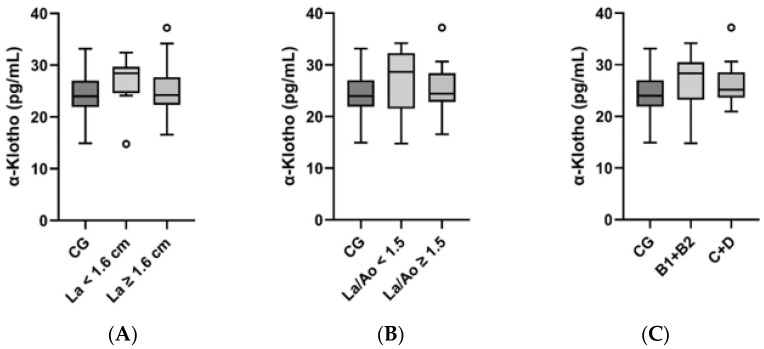
Comparisons for α-Klotho of control group and HCM subgroups ((**A**): La; (**B**): La/Ao; (**C**): staging). Legend: CG, control group; La, left atrium; La/Ao, left atrium/aorta; ○ outlier.

In the general group comparison, no difference could be found between the serum α-Klotho values of the control group and those of the HCM group (*p* = 0.22) (Figure 1). For further analysis, the HCM group was subdivided based on morphological measurements and the ACVIM stages as follows: enlargement of La (normal: <1.60 cm (*n* = 10); enlarged: ≥1.60 cm (*n* = 17)), elevated La/Ao (normal: <1.50 (*n* = 9); elevated: ≥1.50 (*n* = 17); La/Ao value not available for one cat), and ACVIM staging (A–D). ACVIM stages B1 and B2 were summarised as subclinical stages (B1 + B2), and stages C and D comprised animals with previous or acute apparent clinical signs (C + D). Accordingly, there were 13 cats in category B1 + B2 and 14 cats in category C + D. The Kruskal–Wallis-test and post hoc tests did not display any significant differences in α-Klotho concentrations between the subgroups (Figure 2). However, the following was noted for the subdivisions La and La/Ao and for the summarised staging categories: the median α-Klotho concentration was lower in the subgroups and categories comprising more severely diseased subjects (Table 2).

**Figure 3:**
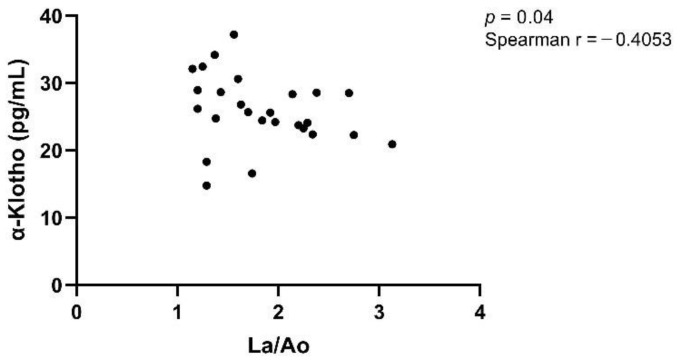
Spearman correlation of α-Klotho and La/Ao. Legend: La/Ao, left atrium/aorta.

The control group had a mean age of 3.8 years (range: 0.5 to 13 years), and the HCM group had a mean age of 8.2 years (range: 2 to 14 years). In both groups, serum α-Klotho concentrations were calculated in correlation (Spearman) to the age of the animals to assess a possible relationship between age and α-Klotho concentration. We could not find such a correlation, neither in the control group (*p* = 0.47, r = −0.1272) nor in the HCM group (*p* = 0.73, r = 0.06873). Furthermore, Spearman’s correlation was calculated for the assessment of potential relationships between α-Klotho and morphological or clinical parameters. No correlations could be detected between the serum α-Klotho concentration and LVWT, La, or ACVIM staging, while there was a significant correlation between the serum α-Klotho concentration and La/Ao (*p* = 0.04, r = −0.4053; Figure 3).

## 4. Discussion

To the authors’ knowledge, this is the first study investigating α-Klotho in feline serum samples. While there are several approaches for addressing Klotho and cardiovascular diseases in experimental and clinical contexts, as well as two studies dealing with Klotho in dogs, this study investigated α-Klotho in cats with HCM. A study by Yi et al. [20] also examined α-Klotho in serum using an ELISA, though this study was on dogs with CKD. This analysis found a difference in the median values between the control group and the CKD group, and the range of values was wider than the one in our study. Both Yi’s study and our study used species-specific assays. Comparing the median and range of healthy dogs in the study from Yi et al. [20] and the healthy cats in our study, we observed higher levels of Klotho in healthy dogs. This is important for calculating reference ranges for Klotho in both species. A study by Semba et al. [17] examined α-Klotho concentrations in plasma using an ELISA in human patients with and without cardiovascular diseases. While the levels of values were quite different in the mentioned studies, it is notable that the control group and the diseased group lay closer together in our study and the mentioned study concerning cardiovascular diseases in humans [17] than they did in the study concerning CKD in dogs [20]. Klotho gene expression mainly takes place in the kidneys and brain, also occurring in various other tissues but to a lower extent [11]. Renal tissue damage, which occurs in acute kidney injury (AKI) and CKD, is associated with reduced blood Klotho levels [25,26]; therefore, Klotho is referred to “[…] as a potential biomarker for […] AKI” [17]. A biomarker, in this sense, is a measurable indicator of a certain disease condition [27]. We did not consider Klotho as a biomarker for diagnosing heart disease in this context. Rather, the aim of our study was to investigate the characteristics of Klotho in HCM as one example for feline cardiomyopathy.

There are several studies dealing with Klotho and cardiac hypertrophy in the context of CKD. LVH, being a part of so-called uremic cardiomyopathy, occurs as a frequent complication of CKD [19]. Yang et al. [19] described a negative relation between an increase in left ventricular mass index and serum Klotho concentrations in human patients with CKD. Xie et al. [28] focused on cardiac hypertrophy and found that there was no difference in the hearts of Klotho-deficient mice and wild type mice, but the Klotho-deficient mice developed more severe cardiac hypertrophy during CKD. A study by Agarwal et al. [29] concerning mice with Klotho deficiency but without CKD supported the thesis that there is no general cardiac hypertrophy concomitant with Klotho deficiency. In our study on patients with primary HCM and without concomitant CKD, no difference in the median α-Klotho concentration between the control group and the HCM group could be revealed. In another study concerning cardiac hypertrophy, Xie et al. [18] discovered an excessive hypertrophic reaction on β-adrenergic overstimulation in Klotho-deficient mice but no general cardiac pathology in those mice. The results from Faul et al. [30] stand in contrast to the assumption mentioned above, as their investigation actually found an increased relative thickness of the left ventricular wall in Klotho-deficient mice without CKD or other diseases. Furthermore, there are studies dealing with Klotho and cardiovascular diseases, e.g., atherosclerosis. A study by Keles et al. [31] found an association between lower Klotho levels in serum and the appearance of atherosclerosis. Semba et al. [17] reported an association of higher Klotho concentrations in plasma with a lower risk of developing cardiovascular diseases such as coronary heart disease or heart failure in a clinical study with human patients. We examined cats with HCM that occurred as a primary disease and not as secondary cardiac hypertrophy. In our analysis, no significant difference in the serum concentrations of α-Klotho in the comparison of the control group versus the HCM group could be detected. It can therefore be assumed that this case of feline heart disease is not a situation derived from systemic Klotho deficiency like the one Hu et al. [25] described kidney disease to be, for example. Klotho was referred to as an agent capable of cardioprotection in some sources [18,32], while it was also stated that Klotho deficiency is not generally responsible for cardiac pathologies [18]. While we did not find any significant difference in Klotho concentrations in the general group comparison, one finding concerning the subdivisions within the HCM group is notable. The median serum concentration for the more severely diseased categories lay lower than the ones for the less diseased categories within La, La/Ao, and the staging subdivisions (B1 + B2, C + D). These differences were not statistically significant, but their setting may give hints that advanced conditions of heart disease may be concomitant with lower Klotho concentrations. Consequently, our results imply that this might be indicated to differentiate between a general group comparison of healthy and diseased groups and comparisons within the diseased group to account for all possible differences. Furthermore, when assessing Klotho in heart diseases, it must be considered whether the heart disease is present alone or in conjunction with other diseases. Our analysis did not show any significant differences in median α-Klotho concentrations between the HCM subgroups (Figure 2). A study investigating serum α-Klotho in the different human CKD stages (stages 1–5) not only found decreased α-Klotho concentrations in the more severe stages of CKD (stages 3, 4, 5) but also found a significant difference between CKD stage 1 and 2. This led to the assumption that Klotho could be suitable as a biomarker for the early stages of CKD [33]. We found a negative correlation between the serum α-Klotho concentrations and La/Ao. The r-value of −0.4053 indicates a rather weak correlation and should be interpreted carefully regarding the other results. Since the left atrium and the La/Ao ratio can be used to assess the disease severity of HCM [34], Klotho could also play a role in this context.

The several limitations of this study must be noted. We measured α-Klotho in feline serum samples. Since one investigation concerning different ELISAs for Klotho quantification mentioned variances in results between plasma and serum concentrations for one assay [35], it must be mentioned that the serum concentrations measured here may differ from similar measurements in plasma. We did not have the opportunity to take measurements in both fluids. Additionally, Heijboer et al. [35] came to the conclusion that some assays in their investigation were lacking accuracy and reliability in terms of their results. To our knowledge, our measurement is the only one of α-Klotho in cats so far, so no comparison can be drawn to other measurements in cats. Furthermore, it was not possible to conduct echocardiography for the cats in the control group. Although these animals did not present with a heart murmur or symptoms for cardiac disease, it has to be mentioned that the eventual presence of HCM could not be ruled out entirely. Analyses with a larger group size, especially for the subgroups, are recommended to validate the results. To verify and expand the knowledge about Klotho in this context, conducting research dealing with the features of Klotho concerning disease prophylaxis and therapy, just as, for example, Hu et al. [36] reported, could also be a promising approach.

## 5. Conclusions

In this study, we assessed α-Klotho in cats, and there was no significant difference in serum concentrations between the healthy control group and the group affected by HCM. The comparison of different disease stages gave hints regarding the possible relations of α-Klotho to the disease stages. Both this fact and the evaluation of a possible association of Klotho in heart diseases with concomitant kidney diseases should be of interest for future studies. Klotho is a biological agent with promising properties and potential applications in disease therapy and prevention, and further investigation into it is warranted in veterinary medicine.

## Figures and Tables

**Table 1:** Descriptive statistics for serum α-Klotho in control group and HCM group.

	Control Group	HCM Group
*n*	34	27
minimum	14.9	14.8
25% percentile	21.9	23.3
median	24.0	25.6
75% percentile	27.0	28.7
maximum	33.2	37.2
mean	24.5	25.8

Values are in pg/mL.

**Table 2:** Median and interquartile ranges for α-Klotho concentrations in control group and HCM group and in HCM subgroups (med [Q1; Q3]).

	α-Klotho (pg/mL)
Control group	24.0 [21.9; 27.0]
HCM group	25.6 [23.3; 28.7]
La < 1.6 cm	28.5 [24.6; 29.7]
La ≥ 1.6 cm	24.2 [22.3; 27.7]
La/Ao < 1.5	28.7 [21.5; 32.3]
La/Ao ≥ 1.5	24.5 [22.8; 28.4]
Category B1 + B2	28.3 [23.2; 30.5]
Category C + D	25.2 [23.6; 28.5]

La, left atrium; La/Ao, left atrium/aorta; med, median; Q1, 25% percentile; Q3, 75% percentile.

## Data Availability

Data are unavailable due to privacy restrictions.
